# Cardiac Fibroblast-Dependent Extracellular Matrix Accumulation Is Associated with Diastolic Stiffness in Type 2 Diabetes

**DOI:** 10.1371/journal.pone.0072080

**Published:** 2013-08-21

**Authors:** Kirk R. Hutchinson, C. Kevin Lord, T. Aaron West, James A. Stewart

**Affiliations:** 1 Department of Physiology, University of Arizona, Tucson, Arizona, United States of America; 2 Feik School of Pharmacy, University of the Incarnate Word, San Antonio, Texas, United States of America; 3 Center for Cardiovascular and Pulmonary Research, Nationwide Children’s Hospital, Columbus, Ohio, United States of America; 4 Department of Biological Sciences, Mississippi State University, Starkville, Mississippi, United States of America; Texas A & M, Division of Cardiology, United States of America

## Abstract

Cardiovascular complications are a leading cause of death in patients with type 2 diabetes mellitus (T2DM). Diastolic dysfunction is one of the earliest manifestations of diabetes-induced changes in left ventricular (LV) function, and results from a reduced rate of relaxation and increased stiffness. The mechanisms responsible for increased stiffness are not completely understood. Chronic hyperglycemia, advanced glycation endproducts (AGEs), and increased levels of proinflammatory and profibrotic cytokines are molecular pathways known to be involved in regulating extracellular matrix (ECM) synthesis and accumulation resulting in increased LV diastolic stiffness. Experiments were conducted using a genetically-induced mouse model of T2DM generated by a point mutation in the leptin receptor resulting in nonfunctional leptin receptors (*db/db* murine model). This study correlated changes in LV ECM and stiffness with alterations in basal activation of signaling cascades and expression of profibrotic markers within primary cultures of cardiac fibroblasts from diabetic (*db/db*) mice with nondiabetic (*db/wt*) littermates as controls. Primary cultures of cardiac fibrobroblasts were maintained in 25 mM glucose (hyperglycemic-HG; diabetic *db/db*) media or 5 mM glucose (normoglycemic-NG, nondiabetic *db/wt*) media. The cells then underwent a 24-hour exposure to their opposite (NG; diabetic *db/db*) media or 5 mM glucose (HG, nondiabetic *db/wt*) media. Protein analysis demonstrated significantly increased expression of type I collagen, TIMP-2, TGF-β, PAI-1 and RAGE in diabetic *db/db* cells as compared to nondiabetic *db/wt*, independent of glucose media concentration. This pattern of protein expression was associated with increased LV collagen accumulation, myocardial stiffness and LV diastolic dysfunction. Isolated diabetic *db/db* fibroblasts were phenotypically distinct from nondiabetic *db/wt* fibroblasts and exhibited a profibrotic phenotype in normoglycemic conditions.

## Introduction

Cardiovascular complications are a leading cause of death in patients with type 2 diabetes mellitus (T2DM). T2DM is an independent risk factor for heart failure and is commonly associated with metabolic syndrome, which includes obesity, dyslipidemia and hypertension. T2DM results in structural and functional abnormalities that ultimately lead to diabetic cardiomyopathy, which is defined as left ventricular (LV) dysfunction independent of coronary artery disease and hypertension [1]. In particular, T2DM closely correlates with heart failure with preserved ejection fraction or diastolic heart failure [2]. Diastolic dysfunction is one of the earliest manifestations of diabetes-induced changes in LV function, and results from a reduced rate of relaxation and increased stiffness. In general, defects in excitation-contraction coupling are thought to underlie relaxation defects [3], [4], while fibrosis, increased collagen accumulation and crosslinking [2], [5], myocyte hypertrophy and titin-isoform switching [6], [7] contributed to the observed changes in diastolic stiffness [8].

The molecular mechanisms responsible for increased LV stiffness in diabetic heart failure are not completely understood. Chronic hyperglycemia, advanced glycation endproducts (AGEs), oxidative stress, activation of the renin-angiotensin system and increased levels of proinflammatory and profibrotic cytokines are molecular pathways known to be involved in regulating LV diastolic stiffness. Indeed, it is most likely the interplay of these pathways that lead to a profibrotic environment within the diabetic myocardium.

Although, interstitial cardiac fibroblasts are known to be key regulators of myocardial remodeling in heart failure, their role in T2DM-induced cardiomyopathy is less well documented. Fibroblasts produce the bulk of the extracellular matrix (ECM) as well as degradative enzymes, such as matrix metalloproteases (MMPs), which regulate and remodel the myocardial ECM, as well as promote collagen synthesis and turnover. In fact, these cells are being recognized as having a number of other functions that directly or indirectly impact myocardial physiology [9]. Several studies have described changes in interactions between fibroblasts and their ECM in response to increased cardiovascular load [10], genetic hypertension [11], pacing-induced heart failure [12] or in response to biochemical factors including angiotensin II (AngII) and transforming growth factor-β (TGF-β) [10], [13]. These studies and others have suggested that alterations in fibroblast function may impact myocardial function.

Recently, the profibrotic effects of culturing normal fibroblasts in high glucose or hyperglycemic levels have been addressed [14]. However, these cells may be phenotypically distinct from cardiac fibroblasts chronically exposed *in vivo* to hyperglycemia and advanced glycation endproducts (AGEs), as is in T2DM. Experiments were conducted using a genetically-induced mouse model of T2DM generated by a point mutation in the leptin receptor gene resulting in nonfunctional leptin receptors (*db/db* murine model). The objective of this study was to compare changes in LV ECM and stiffness and correlate them with alterations in basal activation of signaling cascades and expression of profibrotic markers within primary cell cultures of cardiac fibroblasts from diabetic (*db/db)* mice and nondiabetic (*db/wt*) control littermates. In addition, we sought to determine if changes in glucose concentrations altered basal fibroblast phenotype programming in *db/db* and *db/wt* isolated cells. Our findings demonstrated that T2DM was associated with increased LV collagen accumulation and AGE crosslinking, myocardial stiffness and diastolic dysfunction. This study also revealed that isolated *db/db* fibroblasts were phenotypically distinct from *db/wt* cells and a profibrotic programmed phenotype was maintained despite alterations in media glucose levels.

## Materials and Methods

### Animal Model

All experiments used 15–16 week old male *Lepr^db^* (*db/db*) T2DM mice (BKS.Cg-*Dock7^m^* +/+ *Lepr^db^*/J, Jackson Labs) and the heterozygous (*db/wt*) lean littermates as controls. The *Lepr^db^* leptin receptor mutation is produced by a point mutation in the leptin receptor resulting in nonfunctional leptin receptors insensitive to leptin signaling. These mice develop hyperglycemia by 8-weeks of age, overt diabetes by 12-weeks of age, and exhibit many common features of T2DM including hyperlipidemia, obesity and insulin resistance. The mice were housed under standard environmental conditions and maintained on commercial mouse chow and tap water *ad libitum*. All studies conformed to the principles of the National Institutes of Health “Guide for the Care and Use of Laboratory Animals,” (NIH publication No. 85-12, revised 1996) and the protocol was approved by both the Mississippi State University and Nationwide Children’s Hospital Animal Care and Use Committees. At the 15–16 weeks of age, mice were anesthetized with sodium pentobarbital (50 mg/kg) administered via intraperitoneal injection. At this time, the chest was opened, and the heart was quickly excised for further cellular, histological and biochemical experiments.

### Assessment of LV Function

Mice were anesthetized using a 4% isoflurane gas mixture and then intubated via tracheostomy with a 23 gauge catheter to be subsequently ventilated (isoflurane 2% and oxygen 3 l/min) via Hugo Sachs Elektronic-MiniVent (Harvard Apparatus, type 845). Using an open-chest technique, a 1.4 fr high fidelity pressure-volume catheter (SPR-839; Millar Instruments) was passed through the common carotid artery and aorta and into the LV to record cardiac function. Following a 15-minute equilibration period, baseline cardiac parameters were acquired. Pressure and volume signals were continuously recorded (sampling rate 2000 Hz) using the MPVS-400 P-V conductance system (Millar Instruments). Measures of LV end diastolic pressure were computed using the Millar PVAN analysis system. Preload- and afterload-independent measures of cardiac function such as the end-systolic pressure-volume relationship (ESPVR) and the end-diastolic pressure volume relationship (EDPVR) were assessed by occluding the inferior vena cava to alter preload. For each experimental trial a minimum of three consecutive P-V loops was generated. Data analysis was performed offline (PVAN, version 3.4, Millar Instruments). Echocardiographic measurements (Visual Sonics VEVO 770 Ultrasound System) were also performed prior to conductance catheter analysis as previously described [15]. Once assessments were completed, hearts were excised and either snap-frozen in liquid nitrogen and then stored at −80°C until biochemical analysis could be performed or fixed in 2% paraformaldehyde for histology. An n = 11 mice were used in each group.

### Morphological Analysis of the Collagen Matrix

Myocardial tissue taken from the mid-wall of the LV was fixed in 2% paraformaldehyde and paraffin embedded blocks were prepared. Five micron sections were stained for collagen using picric acid sirius red (F3BA) stain. Estimates of the collagen fractions were obtained by using polarized light and scanning with specific filters for red and yellow (thick) and green (thin) fibers. Pixel quantitation was performed on a minimum of 30–40 myocardial images per animal at 40X magnification. Special care was taken to exclude perivascular and epicardial regions. An n = 11 mice/group were digitized and quantitated using Olympus MicroSuite 5 using color thresholds gated for the following wavelengths red 20–255 nm, green 40–255 nm and blue 35–255 nm.

### Collagen Crosslinking

Myocardial hydroxyproline concentration was determined as described [16]. In brief, 100 mg of cardiac tissue was mixed with 200 µg/mL pepsin (Sigma-Aldrich) in 0.5 M acetic acid and incubated at 37°C with gentle agitation for 24-hours. After 24-hours of pepsin-acetic acid digestion, acid hydrolysis (6 M HCl for 24-hours at 110°C) was performed and collagen crosslinking was determined by measuring hydroxyproline concentration. AGE peptide fluorescence was measured in 24-hours acid hydrolyzed collagen preparations using methodology described by Wróbel et al [17]. To correct for differences in sample collagen content, hydroxyproline measurements and AGE fluorescence intensity were expressed as arbitrary units/mg tissue used for pepsin-acetic acid and HCl hydrolysis.

### Isolation and Culture of Cardiac Fibroblasts

Fibroblasts were isolated from 15–16 week old *db/db* and *db/wt* mice as previously described [18]. Briefly, the mice were sacrificed and the hearts were dissected free from extra-cardiac tissue and atria. Hearts were rinsed in saline solution, minced and digested with collagenase type 2 (Worthington Biochemical). Isolated fibroblasts were then purified by selective attachment to tissue culture plastic. Cells were cultured in Dulbecco’s Modified Eagle’s Medium containing 15% fetal bovine serum, antimycotics and antibiotics and either 25 mM glucose (*db/db* in hyperglycemic media or HG) or 5 mM glucose (*db/wt* in normoglycemic media or NG). Cells were passaged prior to confluency, following detachment with a 0.25% trypsin/0.1% ethylenediaminetetraacetic acid (trypsin/EDTA) solution. Fibroblasts were used prior to passage 2 in all of the described experiments. Cultures were routinely greater than 95% fibroblasts, as assessed by immunocytochemical staining (positive for DDR2). Cells were serum starved for 24 hours, and then exposed to 24-hours of either hyperglycemic (HG) or normoglycemic (NG) media. The experimental groups are as follows: 1) *db/wt* cells in NG media, 2) *db/wt* cells in HG media, 3) *db/db* cells in NG media and 4) *db/db* cells in HG media. Experiments were performed with n = 4–5 separate fibroblast isolations per group.

### Heart and Cardiac Fibroblast Protein Expression

Cells and LV heart tissue samples were extracted in lysis buffer containing 1% Triton X-100, 75 mM NaCL, 5 mM Tris (pH 7.4), 0.5 mM orthovanadate, 0.5 mM EDTA, 0.5 mM EGTA, 0.25% NP-40 and protease inhibitors. Total protein concentrations in each sample were determined using the bicinchoninic acid assay (BCA, Pierce Biotechnology). Equal amounts of lysate (25–50 µg) were separated on a 10% sodium dodecyl sulfate (SDS)-polyacrylamide gels (BioRad Laboratories). Proteins were electrophoretically transferred to nitrocellulose membranes, which were then stained with Ponceau Red to verify even transfer. Membranes were blocked in 5% powdered milk resuspended in TBS and then incubated with the following antibodies: anti-type I collagen (AbCam), anti-TIMP-2 (Chemicon), anti-RAGE (AbCam), anti-PAI-1 (BD Biosceinces), anti-α-smooth muscle actin (Sigma); GAPDH was used to control for loading of cell lysates. Secondary antibodies were goat anti-rabbit or anti-mouse coupled to HRP (Pierce Biotechnology). Blots were developed using SuperSignal reagents (Pierce Biotechnology), exposed to x-ray film, and immunoreactive bands were quantified using an Alpha Innotech gel imaging system. Experiments were performed in duplicate with n = 4–5 separate fibroblast isolations per group.

Accumulation of type I collagen, secretion of PAI-1and TIMP-2 as well as activation of MMP-2 were assayed in conditioned media collected from fibroblasts cultured as described above. Conditioned media were collected and concentrated from 3 mL of media to 500 µL for all samples using MiniCon concentrators (Millipore Corporation). For analysis of type I collagen, PAI-1 and TIMP-2 protein expression in the media, western blot analysis was performed as described above. Corresponding cell lysate loading concentrations were used for conditioned media samples due to phenol red interference in the BCA assay. Western blot membranes were stained with Ponceau Red to confirm even loading and transfer of media samples, and scanned images of Ponceau Red stained blots were used to correct for loading. An arbitrary band at 55–60 kDa was chosen for loading correction. Active MMP-2 was assayed by gelatin zymography (BioRad Laboratories) as previously described [19]. Experiments were performed in duplicate with n = 4–5 sets of separate fibroblast isolations per group.

### TGF-β ELISA

Conditioned media were collected from the above groups and concentrated using MiniCon concentrators (Millipore Corporation). Activated levels of TGF-β1 were determined using a commercially available ELISA kit (R&D Systems) according to the manufacturer’s instructions. Experiments were performed in duplicate with n = 4–5 sets of separate fibroblast isolations per group.

### Reverse Transcription-Polymerase Chain Reaction (RT-PCR)

Total RNA was isolated from cardiac fibroblasts using RNeasy Cleanup kit (Qiagen). cDNA synthesis was performed using iScript cDNA Synthesis Kit (Bio-Rad) as per manufacturer’s instructions [19]. qPCR was performed as previously described [20]. Briefly, RT-PCR was done with 2 µg total RNA and primers specific for mouse type 1 collagen and mouse β-actin for normalization (primer sequence given in the table below). Preliminary amplification experiments (15–35 cycles) were performed to determine early logarithmic phase of amplification for each mRNA of interest. Relative RNA levels are expressed as the type I collagen to β-actin ratio. Experiments were performed in duplicate with n = 4–5 sets of separate fibroblast isolations per group.

Type I Collagen Forward Primer 5′-GAGCGGAGAGTACTGGATCG-3′

Type I Collagen Reverse Primer 5′-GTTCGGGCTGATGTACCAGT-3′

β-Actin Forward Primer 5′-AGAGGGAAATCGTGCGTGAC-3′

β-Actin Reverse Primer 5′-CAATAGTGATGACCTGGCCGT-3′

### Statistical Analysis

For LV morphological, physiological data as well as collagen volumes, analysis of variance (ANOVA) was performed followed by Tukey’s test of multiple comparisons using GraphPad Prism 4 software to test for statistical differences, defined as p<0.05. For basal fibroblast protein expression, individual experiments were performed from 4–5 separate fibroblast isolations and unpaired Student’s *T*-test was performed using GraphPad Prism 4 software to test for statistical differences between groups, defined as p<0.05. Error bars represent ± standard error of the mean (SEM).

## Results

### T2DM Alters LV Structure and Decreases LV Function

To evaluate the effects of diabetes on LV morphology and physiology, 11 age-matched mice per group were used to perform independent evaluations. Diabetic (*db/db*) mice displayed elevated body weights as well as marked hyperglycemia, but decreased heart weight/tibia length ratios compared to nondiabetic (*db/wt*) mice (Table 1). Echocardiographic analysis confirmed decreased heart size in *db/db* mice as seen in significantly decreased LV end-diastolic dimensions (Table 1). The differences in *db/db* and *db/wt* hearts can be visually observed (Table 1a). Functional analysis with pressure catheters indicated no differences in systolic function (data not shown) between animal groups, however a significant increase was reported in the slope demarcating the end diastolic pressure-volume relationship (EDPVR) in *db/db* hearts (0.130±0.030) compared to *db/wt* hearts (0.053±0.005) (Table 1b). This latter measurement serves as an indicator of a stiffer, less compliant LV which is possibly due to increases in matrix production or ECM accumulation.

**Table 1:** Morphological and Physiological Analysis.

Experimental Groups	Nondiabetic db/wt mice	Diabetic db/db mice
**Body weight (g)**	31.81±0.63	55.16±0.72*
**Heart weight (mg)**	175.0±17.9	152.3±14.5
**Tibia length (mm)**	17.80±1.17	18.61±1.03
**HW/tibia length ratio**	0.0099±0.0011	0.0082±0.0009*
**Glucose (mg/dL)**	164±6	508±26*
**LV EDD (mm)**	4.05±0.28	3.75±0.31*
**EDPVR**	0.0534±0.005	0.13±0.030*

Morphological and physiological data from nondiabetic (*db/wt*) and diabetic (*db/db*) mouse hearts. **Table 1a**. Morphological and physiological data are presented in the table. HW, heart weight; tibia length; HW/tibia length ratio, blood glucose levels, and LV EDD (left ventricular end diastolic diameter) are shown. (*p<0.05 vs *db/wt)* Data represents an n = value 11 mice per group **Table 1b**. Picture inset visually demonstrates differences in heart morphology of diabetic *db/db* mouse (right) and nondiabetic *db/wt* mouse (left). **Table 1c** **.** End diastolic pressure volume relationships (EDPVRs) are significantly increased in diabetic (*db/db*) hearts (right) indicating a stiffer less compliant left ventricle (LV) compared to nondiabetic (*db/wt*) control hearts (left).

### T2DM Increases Collagen Accumulation and Crosslinking

**Figure 1:**
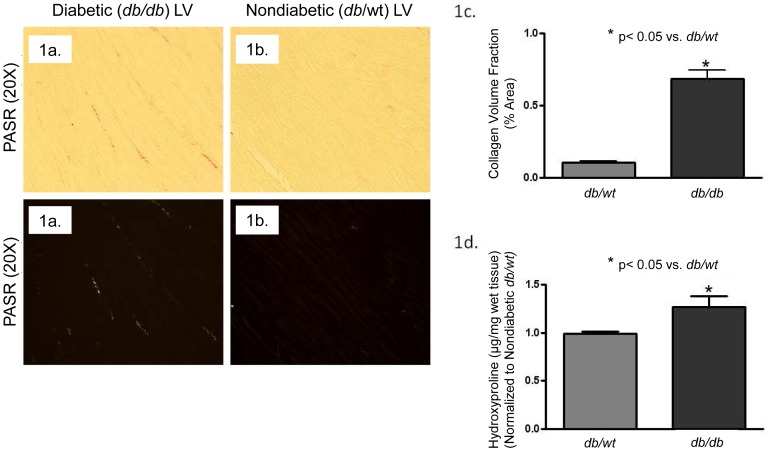
LV collagen volume fractions and collagen crosslinking was increased in diabetic (*db/db)* mouse hearts. LV collagen volume fractions were determined from picric acid sirius red (PASR) stained mid ventricle for non-diabetic (*db/wt*) and diabetic (*db/db*) mice. **Figure 1a** shows representative photographs of PASR stained diabetic (*db/db*) mouse hearts under brightfield and polarized light microscopy. **Figure 1b** shows representative photographs of PASR stained of non-diabetic (*db/wt*) mouse hearts under brightfield and polarized light microscopy. **Figure 1c** depicts a significant increase in PASR stained collagen fibrils in *db/db* LVs (*p<0.05; n = 11 hearts per group). LV collagen crosslinks were determined from subsequent digestions of pepsin-acetic acid and HCl hydrolysis. **Figure 1d** depicts a significant increase in crosslinked collagen in *db/db* as measured by hydroxyproline concentrations. NG = normoglycemic media; HG = hyperglycemic media (*p<0.05; n = 11 hearts per group).

To examine gross changes in LV collagen matrix, prepared slides of paraformaldehyde-fixed LV mid-wall sections from each experimental animal used for functional analysis, were stained with picric acid sirius red (F3BA), photographed and pixel count was quantitated to determine changes in LV collagen volumes. Diabetic (*db/db*) LVs (Fig. 1a) had significantly higher levels of interstitial collagen than nondiabetic (*db/wt*) LVs (Fig. 1b). In fact, there was a 58% increase in stained collagen fibrils in *db/db* LVs (Fig. 1c) (*db/db* 0.69±0.06) compared to. *db/wt* LVs (0.11±0.03). Additionally, changes in collagen crosslinking assessed by measuring hydroxyproline levels from the acid hydrolyzed insoluble fraction of LV myocardium. *db/db* hearts (1.27±0.11) showed significantly increased hydroxyproline levels as compared to *db/wt* hearts.

### T2DM Stimulates Basal Fibroblast Protein Expression

**Figure 2:**
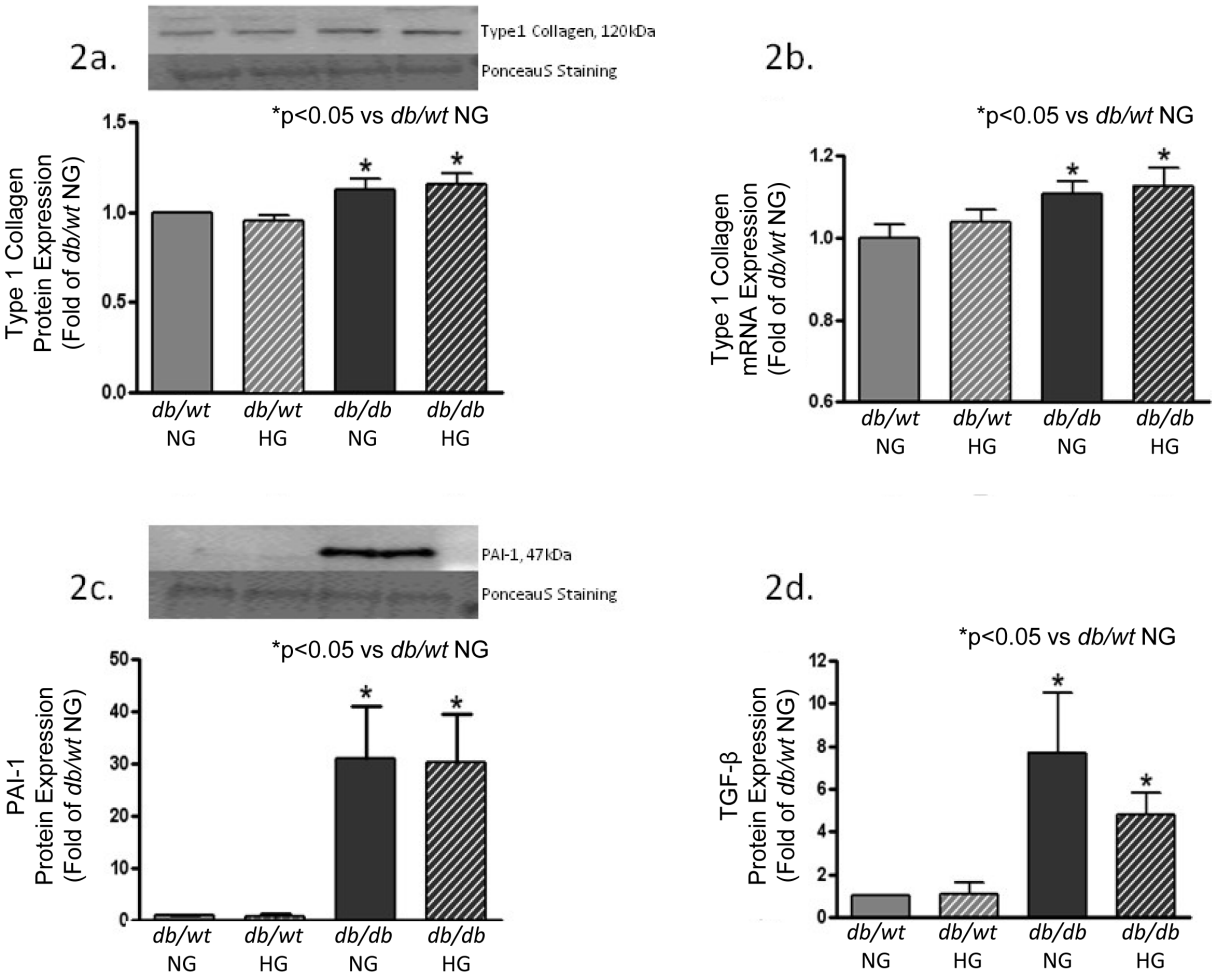
Profibrotic markers were increased in isolated diabetic (*db/db*) cardiac fibroblasts. **Figure 2a** shows type I collagen was increased in isolated fibroblasts from diabetic (*db/db*) protein secreted into conditioned media and in **Figure 2b**. type 1 collagen mRNA expression. **Figure 2c** shows an increased PAI-1 protein expression from cardiac fibroblast conditioned media of diabetic (db/db) fibroblasts. **Figure 2d** demonstrates an increase in TGF-β protein expression in diabetic (*db/db*) from cardiac fibroblast conditioned media as measured by commercial ELISA kit. Smooth muscle expression did not change between groups (data not shown). NG = normoglycemic media 5 mM glucose; HG = hyperglycemic media 25 mM glucose (*p<0.05; n = 4–5 independent experiments with 6 hearts per experiment).

**Figure 3:**
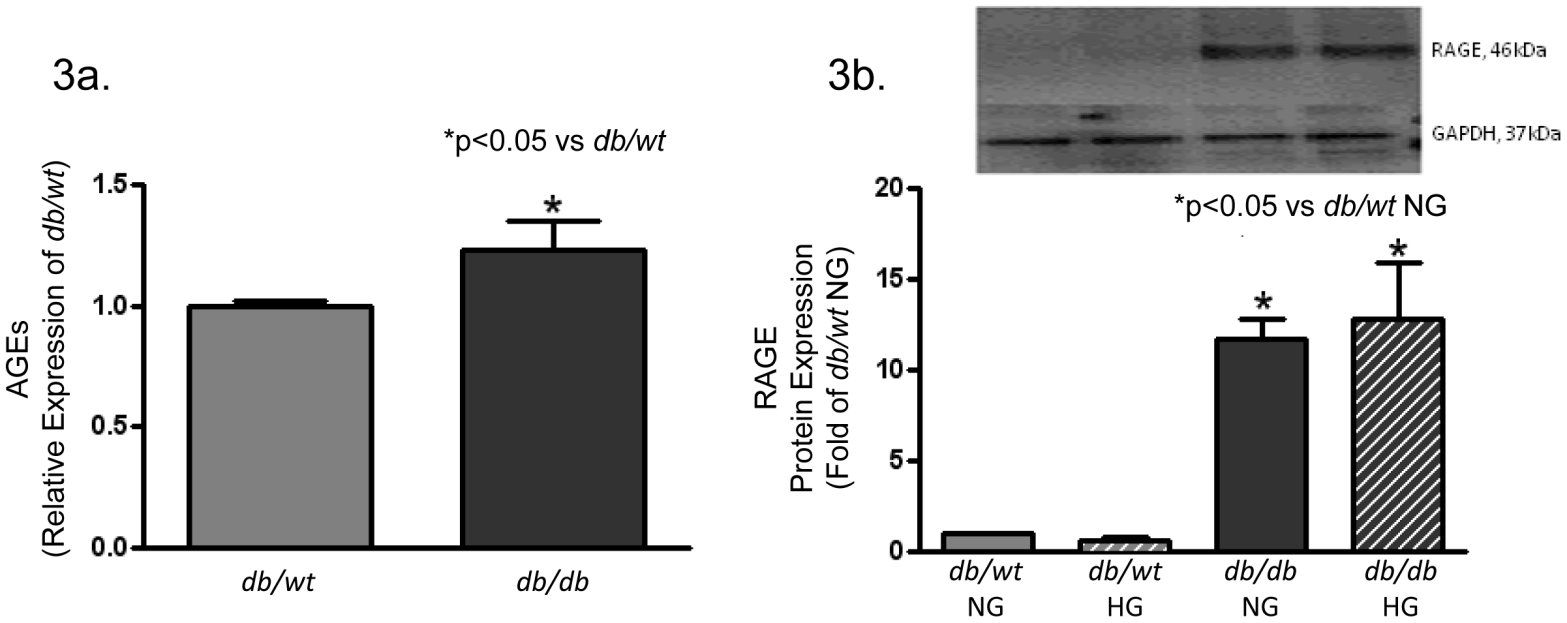
AGE accumulation was increased in diabetic (*db/db*) mouse hearts, and RAGE expression was increased in diabetic (*db/db*) isolated fibroblasts. **Figure 3a**. demonstrates an increased AGE expression in diabetic (*db/db*) LV collagen extracts. **Figure 3b**. depicts an increase in RAGE protein expression in isolated *db/db* LV fibroblasts. NG = normoglycemic media 5 mM glucose; HG = hyperglycemic media 25 mM glucose (*p<0.05; n = 4 independent experiments with 6 hearts per experiment).

To evaluate changes in cell phenotype, cardiac fibroblasts were isolated from diabetic (*db/db*) and nondiabetic (*db/wt*) mouse hearts. Glucose levels in the media were acutely altered for 24-hours to determine if exposure to hyperglycemic or normoglycemic conditions promoted alterations in basal cardiac fibroblast protein expression. Changes from a normoglycemic (NG) to hyperglycemic (HG) environment or vice versa failed to yield significant modifications in basal fibroblast protein expression in either *db/db* or *db/wt* isolated cells, and data for both glucose exposure conditions are presented in the corresponding figures. Basal secretion of type 1 collagen from isolated *db/db* fibroblasts was 15% higher that of *db/wt* isolated fibroblasts (Fig. 2a). These changes were concomitant with significant increases in type I collagen mRNA levels (12%) (Fig. 2b), as compared to *db/wt* cells. While these differences are not overwhelming, changes in type 1 collagen protein and mRNA expression are indicative of a basal diabetic profibrotic phenotype being maintained *ex vivo*. Despite acute exposure of *db/db* fibroblasts to NG media or *db/wt* fibroblasts to HG media, there was no change in type 1 collagen protein expression. Changes in basal levels of collagen production can be triggered by multiple profibrotic stimulators, such as plasminogen activator inhibitor (PAI-1) and transforming growth factor-β (TGF-β) [21]–[23]. Secretion of plasminogen activator inhibitor-1 (PAI-1), a protease inhibitor important for down-regulation of plasmin and fibrin proteolysis [24], [25], was significantly elevated in *db/db* conditioned media as compared to *db/wt* conditioned media (Fig. 2c). Acute exposure of *db/db* cells to NG media or *db/wt* cells to HG media did not alter PAI-1 protein expression. To assess TGF-β levels, a commercially available ELISA was used to measure TGF-β in conditioned media. There was a significant increase (5–7 fold) in TGF-β expression in conditioned media from *db/db* cardiac fibroblasts compared to that of *db/wt* cells (Fig. 2d). There were no changes in TGF-β protein expression in *db/db* fibroblasts acutely exposed to NG media or *db/wt* fibroblasts acutely exposed to HG. AGE formation and accumulation due to chronic hyperglycemia are common complications in T2DM, and they are associated with increased collagen crosslinking [26]. Increased RAGE levels have also been strongly implicated in the pathogenesis of renal and cardiovascular complications [26] [27]. In this study we observed a significant increase in both basal AGE expression in the diabetic heart (Fig. 3a) as well as RAGE expression in *db/db* fibroblasts (Fig. 3b) as compared to their *db/wt* controls. RAGE expression levels did not change when *db/db* cells were exposed to NG media or when *db/wt* cells exposed to HG.

**Figure 4:**
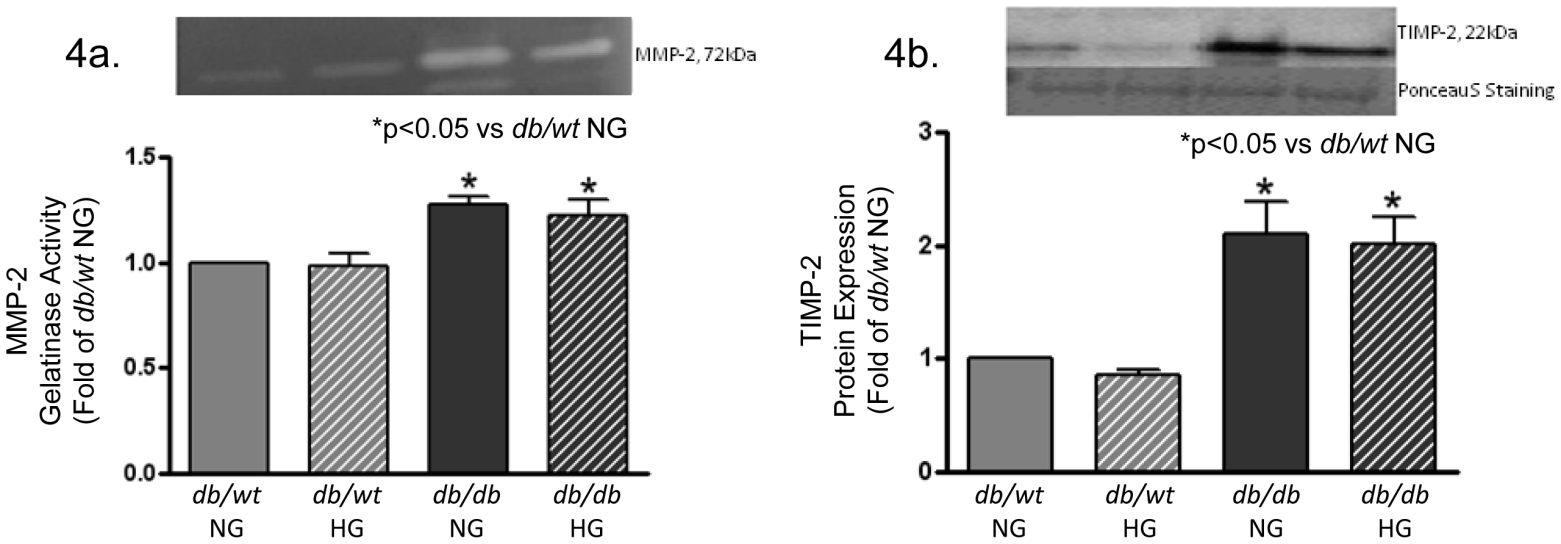
Extracellular matrix protein regulators MMP-2 and TIMP-2 were increased in diabetic (*db/db*) mouse hearts and conditioned media from isolated diabetic (*db/db*) cardiac fibroblasts. **Figure 4a** demonstrates increased MMP-2 gelatinase activity levels from conditioned media of isolated *db/wt* and *db*/db hearts as measured by gelatin zymography. MMP-2 activity levels were also significantly increased in *db/db* LV lysates as measured by gelatin zymography (data not shown). **Figure 4b** shows a dramatic increase in TIMP-2 protein levels for isolated *db/db* cardiac fibroblasts. NG = normoglycemic media 5 mM glucose; HG = hyperglycemic media 25 mM glucose (*p<0.05; n = 4 independent experiments with 6 hearts per experiment).

Zymogram analysis of MMP-2 gelatinase, an important matrix metalloprotease produced by cardiac fibroblasts to degrade collagen and gelatin, was performed using extracted conditioned media from cardiac fibroblast cultures (Fig. 4a). There was approximately a 20% increase in *db/db* gelatinolytic activity, which was significantly higher than that of *db/wt* samples. This increase in MMP-2 activity was mirrored in LV tissues (data not shown). Changing glucose concentration levels had no significant effect on MMP-2 activation in either *db/db* cells or *db/wt* cells. This was contrary to previous studies using adult renal fibroblasts as well as adult cardiac and adventitial fibroblasts [14], [28] which demonstrated changes in MMP activity and expression with changes in glucose levels. Tissue inhibitor of matrix metalloproteases-2 (TIMP-2) secretion was also analyzed. TIMPs, which will non-covalently complex with latent zymogens to suppress MMP activation, were significantly elevated in conditioned media from *db/db* cardiac fibroblasts as compared to media from *db/wt* cells (Fig. 4b). TIMP-2 expression was also not altered regardless of glucose conditions.

Lastly, changes in α-smooth muscle actin (α-SMA) expression were used to correlate increases in basal protein production in diabetic fibroblasts and to mark myofibroblast phenotype differentiation, particularly those exposed to hyperglycemic conditions (data not shown). Myofibroblasts have been described as a stressed fibroblast with enhanced secretory and contractile properties [29]–[31]. Western blots of fibroblasts cultured for 24-hours in different glucose conditions showed α-SMA expression was not altered in *db/db* cells in NG media or *db/wt* cells in HG media.

## Discussion

Cardiomyopathy, accompanied by increased myocardial fibrosis, has been a noted complication for diabetic patients [1]. Termed diabetic cardiomyopathy, this condition is described as one in which diabetes exerts direct pathological effects on the heart without other confounding complications, such as hypertension [32]. In fact, 40%–50% of diabetic patients, without pre-existing cardiovascular disease, have reported LV end diastolic abnormalities [14]. van Hoeven and Factor advanced this concept that diabetic patients had decreased ventricular function due to increased matrix accumulation [33]. Considering alterations in myocardial structure and function can be caused by factors affecting interstitial fibrosis, the role of fibroblasts in collagen synthesis and deposition is critical to understanding the progression of diabetic cardiomyopathy. This study was designed to investigate changes in LV ECM and stiffness and to correlate them with alterations in basal activation of signaling cascades and expression of profibrotic markers within primary cell cultures of cardiac fibroblasts from diabetic (*db/db*) and nondiabetic (*db/wt*) control littermates. In addition, we sought to determine if acute changes in glucose concentration would alter basal fibroblast phenotype programming in *db/db* and *db/wt* isolated cells.

To determine if morphological changes in the diabetic heart affected LV physiological performance, conductance catheter studies were performed to determine LV end diastolic pressure volume relationships (EDPVRs) in both *db/db* and *db/wt* animals. LV EDPVRs were shown to be significantly higher in the *db/db* mouse hearts as compared to the *db/wt* controls. This data indicates that *db/db* hearts had decreased ventricular compliance. Diastolic dysfunction has been correlated with increased collagen deposition in multiple experimental models of diabetes [34], [35], and limiting pathological matrix accumulation attenuates cardiac remodeling improving ventricular function [36]. In this study, *db/db* hearts had increased collagen myocardial deposition with elevated collagen crosslinks as indicated by hydroxyproline content. Our results confirm that T2DM is associated with a profibrotic environment that alters ventricular performance. Dramatic changes in ECM accumulation under pathological conditions have primarily been demonstrated to be regulated by cardiac fibroblasts [31], [37].

Cardiac fibroblasts are one of the most populous cell types in the heart, second only to cardiac myocytes, and they are responsible for normal maintenance and pathological remodeling of the myocardial ECM [19], [38]. Cardiac fibroblasts interact with the surrounding ECM and are integral to the organization of tissue architecture and support of basic cellular functions, including survival, proliferation and migration [39]. Accordingly, increased collagen production would not only be expected to cause structural rearrangements in the myocardium, but also modifications in fibroblast-ECM interactions. These modifications often correspond with changes in cellular phenotype. For example, cardiac fibroblasts isolated from animals with hypertension as well as those isolated from sites of myocardial infarction demonstrate enhanced contractility, decreased migration, and increased collagen production and accumulation [11], [40], [41]. Increases in matrix protein accumulation and deposition may be the result of either enhanced production and/or decreased degradation of the collagenous ECM. Our experimental data supports the causative role of altered collagen turnover as assessed through changes in type I collagen protein and mRNA levels, which were significantly up-regulated in *db/db* cardiac fibroblasts. It is important to note that type I collagen mRNA and protein expression was the only collagen type measured *in vitro*. Picric acid sirius red stained LV tissue sections from diabetic hearts demonstrated a much higher collagen content and this elevated level cannot be directly correlated to increases in type 1 collagen alone. Interestingly, when glucose media levels were changed, the protein and mRNA expression of type 1 collagen was not altered for either *db/db* or *db/wt* fibroblast cultures. These results differ from those of previous studies, whereby there was a concomitant increase in collagen with increased glucose concentrations *in vitro* [14], [42]. The profibrotic changes could be attributed to either to species differences (rat vs. mouse), diabetic animal model variances (experimental vs. genetic), or a time dependent exposure to hyperglycemic growth media conditions. The paucity of clinical and animal research data using myocardial fibroblasts may explain the scientific disparities observed in the literature. More extensive studies are needed to determine the significance of fibroblast functional changes in diabetic animals, as this represents a potential mechanism of diabetes-related myocardial dysfunction. This observation is also intriguing because it establishes the possible existence of a programmed, profibrotic cellular phenotype that is “primed” by elevated diabetes-related biochemical factors within the cell’s local environment.

T2DM is a multifaceted disease, in which mechanical and biochemical stimuli have been shown to modulate the interactions between fibroblasts and ECM [10], [13]. Of these, TGF-β is associated with a profibrotic signaling cascade with resultant activation of SMAD 2/3 leading to increased myocardial fibrosis and increased ventricular stiffness [23]. In this study, diabetic *db/db* fibroblasts secreted significantly higher levels of TGF-β than nondiabetic *db/wt* as measured by ELISA. Contrary to earlier findings [28], exchanging glucose levels failed to significantly effect TGF-β expression in either *db/db* or *db/wt* cells. Similar findings by Solini et al. showed TGF-β mRNA transcript levels were not changed by altering glucose levels in a human fibroblast culture model system [43]. PAI-1 is another potent, biochemical stimulus capable of contributing to a profibrotic environment by inactivating proMMPs [44]. Our data demonstrated significantly higher levels of PAI-1 in *db/db* cell culture media than that of *db/wt* cell media. It has been reported that in fibrotic tissues PAI-1 concentrations are significantly elevated, and increased PAI-1 levels have been noted to limit MMP degradation activities [44] [45]. In this study we defined that when cardiac fibroblasts are removed from a T2DM-mediated continuum of mechanical and chemical stressors, they will maintain a profibrotic, diabetic culture environment. This diabetic setting may contribute to a programmed cell phenotype, which we have observed in this study. Altering glucose levels acutely did not affect the overall basal cellular response. In fact, chronic exposure to T2DM *in vivo* resulted in an increased profibrotic cellular protein profile in *db/db* fibroblasts compared to that of *db/wt* levels.

It has been well documented in hyperglycemic conditions long-lived structural proteins, such as collagen, can be nonenzymatically modified by advanced glycation end products (AGEs) [31]. These modified proteins not only stiffen the ECM by increasing collagen network crosslinks, but also AGEs serve as an agonist to receptors for AGE (RAGE). AGEs are formed when increased levels of glucose, as found in T2DM, react non-enzymatically with proteins to form reversible Schiff bases and then Amadori compounds [46]. These Amadori compounds undergo further chemical modifications to become irreversibly crosslinked derivatives or AGES [46]. AGEs also occur normally with age under normoglycemic conditions; however under the hyperglycemic conditions common in diabetics, the rate of AGE formation is enhanced and impairment of cardiac function occurs at even earlier rate than found in the nondiabetic population [46]–[48]. Clinically, diabetic patients have significantly higher AGE accumulation and interstitial fibrosis resulting from chronic hyperglycemia [2]. RAGE is a multi-ligand receptor that is normally expressed at low levels; however, its expression is increased during aging and in response to diabetes [2], [46]. AGE/RAGE activation stimulates the secretion of numerous profibrotic growth factors as well as promotes collagen deposition leading to tissue fibrosis [27]. These changes in AGE accumulation, RAGE activation and resulting fibrosis have been closely associated with increased diastolic LV stiffness [2]. In this study, AGE levels were elevated in both diabetic *db/db* and nondiabetic *db/wt* mouse hearts, but they were only significant in the *db/db* hearts. AGE accumulation in *db/wt* cells occurs normally as an individual ages [47], [48], however *db/wt* cells failed to upregulate RAGE. Consequently, in *db/db* myocardial stiffening increased as collagen-AGE crosslinks occurred. RAGE expression appears to be a prominent aspect in diabetes- mediated ECM remodeling. In this study increasing AGE/RAGE signaling promoted myocardial fibrosis, as well as altered the mechanical properties of the ECM resulting in diastolic dysfunction.

The balance between ECM synthesis and degradation is thought to be a tightly regulated in the heart, where increases or decreases in either MMPs or TIMPs could result in dynamic changes in the myocardial matrix resulting in alterations in cardiovascular function. The data from this study demonstrated increased MMP-2 activity from both whole heart and isolated fibroblast extractions from *db/db* samples. In addition, TIMP-2 expression was significantly elevated in isolated *db/db* fibroblasts. It has been reported that plasma and urine samples from human diabetic patients had significantly increased MMP-2/-9 and TIMP-1/-2 levels [49], [50]. Their presence reflects a dysregulation in matrix metabolism that is indicative of an ECM imbalance between MMP-2-mediated collagen degradation and TIMP-2-mediated MMP inhibition. The resulting outcome that was reported was an increase in matrix protein synthesis and accumulation similar to T2DM [51]. This observation offers a mechanism found in diabetic animals in which impaired collagen turnover contributes to increased matrix deposition and accumulation. This matrix will remain longer and become more crosslinked to mechanically stiffen the cell’s microenvironment. Currently, our laboratory is investigating if changes in AGE-collagen crosslinks prevent MMP-mediated ECM degradation or if increased AGE/RAGE signaling promotes TIMP expression.

Leptin is a cytokine that when released from connective tissue cells, like adipocytes, will act on its receptor to affect body weight homeostasis [52]. The leptin receptor has been grouped into the class I cytokine receptor superfamily because of shared homology with a number of mitogenic and proliferative signal transducers [52]. In this study the *db/db* leptin receptor deficient murine model was used to determine LV functional alterations and fibroblast-mediated changes occurring as a result of T2DM. There is existing evidence that animal models with either a leptin deficiency or a leptin receptor deficiency are protected from fibrosis [53]–[55]. Our data clearly demonstrates that *db/db* hearts express significantly more collagen that their *db/wt* controls, and the diabetic fibroblast profibrotic setting is being maintained *in vitro*.

The data presented within this study demonstrated a correlation exists between LV ECM stiffness, fibrosis, AGE-mediated collagen crosslinks and alterations in basal expression of profibrotic markers. Collectively, these factors will create a stiffer myocardium that will alter diabetic (*db/db*) LV compliance as compared to nondiabetic (*db/wt*) controls. These external factors will perpetuate a diabetes-programmed fibroblast phenotype. Additionally, this study showed that acute changes in glucose concentrations did not alter the basal fibroblast phenotype *in vitro*. While this data may be contrary to previous studies, we presented an alternative hypothesis that a diabetic cellular programming may exist in *db/db* fibroblasts, and this setting cannot be overridden by acute changes in glucose concentrations. The basal fibroblast response to T2DM is responsible for activating signaling cascades to increase expression of profibrotic markers within the myocardium of *db/db* hearts. While these results depict a relevant pathological response, additional data will still need to be gathered to fully understand the fibroblast behavior for better insight into the pathological effects of diabetic cardiomyopathy.
